# Using respiratory challenges to modulate CSF movement across different physiological pathways: An fMRI study

**DOI:** 10.1162/imag_a_00192

**Published:** 2024-06-10

**Authors:** Vidhya Vijayakrishnan Nair, Tyler C. Diorio, Qiuting Wen, Vitaliy L. Rayz, Yunjie Tong

**Affiliations:** Weldon School of Biomedical Engineering, Purdue University, West Lafayette, IN, United States; Department of Radiology and Imaging Sciences, Indiana University School of Medicine, Indianapolis, IN, United States

**Keywords:** cerebrospinal fluid, paced breathing, breath holding, fMRI, hemodynamics, low-frequency oscillations

## Abstract

With growing evidence signifying the impact of cerebrospinal fluid (CSF) flow in facilitating waste clearance from the brain and potential pathophysiological links to neurodegenerative disorders, it is of vital importance to develop effective methods to modulate CSF flow in the brain. Here, we attempt this by means of simple commonly used respiratory challenges—paced breathing and breath holding. Functional Magnetic Resonance Imaging scans of the brain and neck respectively were used to record the craniad and caudad CSF movements at the fourth ventricle from eight healthy volunteers during paced breathing and breath holding. Further, we utilized a novel approach for the first time to combine these separately acquired unidirectional CSF movement signals to compare the CSF flow in both directions (in the fourth ventricle) with the respiratory stimuli as a physiological control. Our results demonstrate that these respiratory challenges enhance the magnitude as well as control the direction of CSF movement in the fourth ventricle. They also reveal the capability of blood CO_2_concentration changes (induced by respiratory challenges) in the low-frequency range to bring about these CSF movement modulations. Finally, we also successfully report our novel approach where we use these breathing challenges as a unique control condition to detect the small net CSF flows from independently captured unidirectional signals.

## 1 Introduction

Cerebrospinal fluid (CSF) movement within the brain ventricles and subarachnoid spaces of the cranium and the spine is critical to the health and function of the central nervous system (CNS). Besides delivering nutrients, hormones, and immune system components throughout the CNS, it also provides mechanical protection for the brain and spinal cord (Schwartz & Baruch, 2012;Spector & Johanson, 2007;Spector et al., 2015). Additionally, recent studies have highlighted the role of CSF movement in the pathophysiology of neurodegenerative disorders (Braun & Iliff, 2020;Simon & Iliff, 2016) and the glymphatic system (Jessen et al., 2015). According to current theories on the glymphatic system, a perivascular macroscopic convective fluid transport network that removes soluble proteins and metabolites from the CNS, the movement, circulation, and further exchange of CSF with the interstitial fluid (ISF) are crucial to eliminating neuro-metabolic wastes (Reddy & van der Werf, 2020).

However, in contrast with blood circulation, CSF has no known ‘engine’ to propel its movement through the CNS pathways. In fact, CSF movement within the CNS has been shown to be driven by different physiological forces. For instance, intrathoracic pressure changes associated with respiration have been demonstrated as a possible ‘driver’ of CSF in humans by several Magnetic Resonance Imaging (MRI) studies (Aktas et al., 2019;Dreha-Kulaczewski et al., 2015,^2017^;Friese et al., 2004;Klose et al., 2000;Vijayakrishnan Nair et al., 2022). Similarly, the slow volume changes in blood vessels referred to as vascular low-frequency oscillations (LFOs: 0.01–0.1 Hz), have been demonstrated to play a dominant role in driving CSF movement at the fourth ventricle in humans. This close coupling relationship between changes in LFOs in the cerebral circulation and CSF movement at the fourth ventricle has been illustrated by multiple functional MRI (fMRI) studies, during both resting-state wakefulness and sleep, among young, healthy adults (Fultz et al., 2019;H. C. Yang et al., 2022;Picchioni et al., 2022;Vijayakrishnan Nair et al., 2022,^2023^). Our recent fMRI study has also shown that there exists a cross-frequency interaction between these two separable pathways controlling CSF movement, potentially through blood CO_2_concentration changes (Vijayakrishnan Nair et al., 2022). Moreover, controlled inhalation of a higher concentration of CO_2_has also been recently illustrated to impact LF CSF movement in the fourth ventricle (van der Voort et al., 2024).

Considering the significance of CSF flow dynamics in facilitating brain waste clearance, the imperative lies in devising uncomplicated yet impactful interventions. By enhancing CSF flow dynamics, it might be plausible to potentially impede the accumulation of neuro-metabolic wastes and even influence the prognosis of neurodegenerative conditions such as Alzheimer’s disease (AD) (Li et al., 2022). Respiratory challenges such as paced breathing and breath holding are commonly used to elicit changes in cerebrovascular oscillations in the LF range through changes in the concentration of blood CO_2_. Briefly, breath holding leads to a build-up of CO_2_(i.e., hypercapnia) and consequent cerebral vasodilation (Bright & Murphy, 2013;Larsen et al., 2021;Magon et al., 2009;Raitamaa et al., 2019;Thomason et al., 2005), whereas paced breathing decreases blood CO_2_concentrations leading to transient hypocapnia and cerebral vasoconstriction (Bright et al., 2009;Posse et al., 1997;Sousa et al., 2014;Vogt et al., 2011). However, the effects of these stimuli as potential pathways to manipulate CSF movement oscillations indirectly through alterations in cerebrovascular tone have not yet been explored. Moreover, intrathoracic pressure changes during paced respiration have been shown to directly affect the large draining veins from the brain and result in internal pressure changes in the brain and thereby drive CSF movement within the specific frequency range of paced or free breathing (Dreha-Kulaczewski et al., 2017;Vijayakrishnan Nair et al., 2022). Taken together, these simple respiratory challenges present unique pathways for modulating CSF movement. Therefore, the primary goal of the present study is to evaluate these simple respiratory challenges such as paced breathing and breath holding as potential mechanisms to enhance the magnitude of CSF movement oscillations in specific directions.

The existing fMRI-centered approach exclusively records cerebrospinal fluid motion in a singular direction, either towards the cranium (craniad) or away from it (caudad), contingent on the positioning of the scan volume. This circumstance prohibits the evaluation of overall cerebrospinal fluid flow. Therefore, an additional goal of the current study is to overcome this limitation and reconstruct net CSF movement signals through the use of respiratory challenges during the fMRI brain and neck scans. These challenges act as a unique physiological control condition bridging the unidirectional CSF signals independently captured from the brain and neck scans (under the same respiratory challenge condition) and thereby allow us to stitch these craniad and caudad CSF movements respectively into net biphasic CSF movement signals. Furthermore, we also employ a recently developed methodology from our group (Diorio et al., 2023) to quantify net biphasic CSF velocity profiles during the breathing challenges.

In short, the present study aims to investigate the following: (1) The ability of respiratory challenges—paced breathing and breath holding as unique physiological vasoactive stimuli—to modulate the magnitude and directionality of CSF movement in the human brain (indexed at fourth ventricle) and (2) A novel methodology to recover biphasic CSF velocity waveforms in vivo using a combination of fMRI scans and respiratory challenges.

## 2 Methods

### 2.1 Participants

Eight healthy participants (4 females) aged 19–48 (25.75 ± 9.53) years were included in this study, approved by Purdue University’s Institutional Review Board. Written informed consent was obtained from all participants before the experiments.

### 2.2 Experimental design

An illustrative overview of the experimental design and biphasic CSF velocity calculation paradigm is provided inFigure 1.

**Fig. 1.**
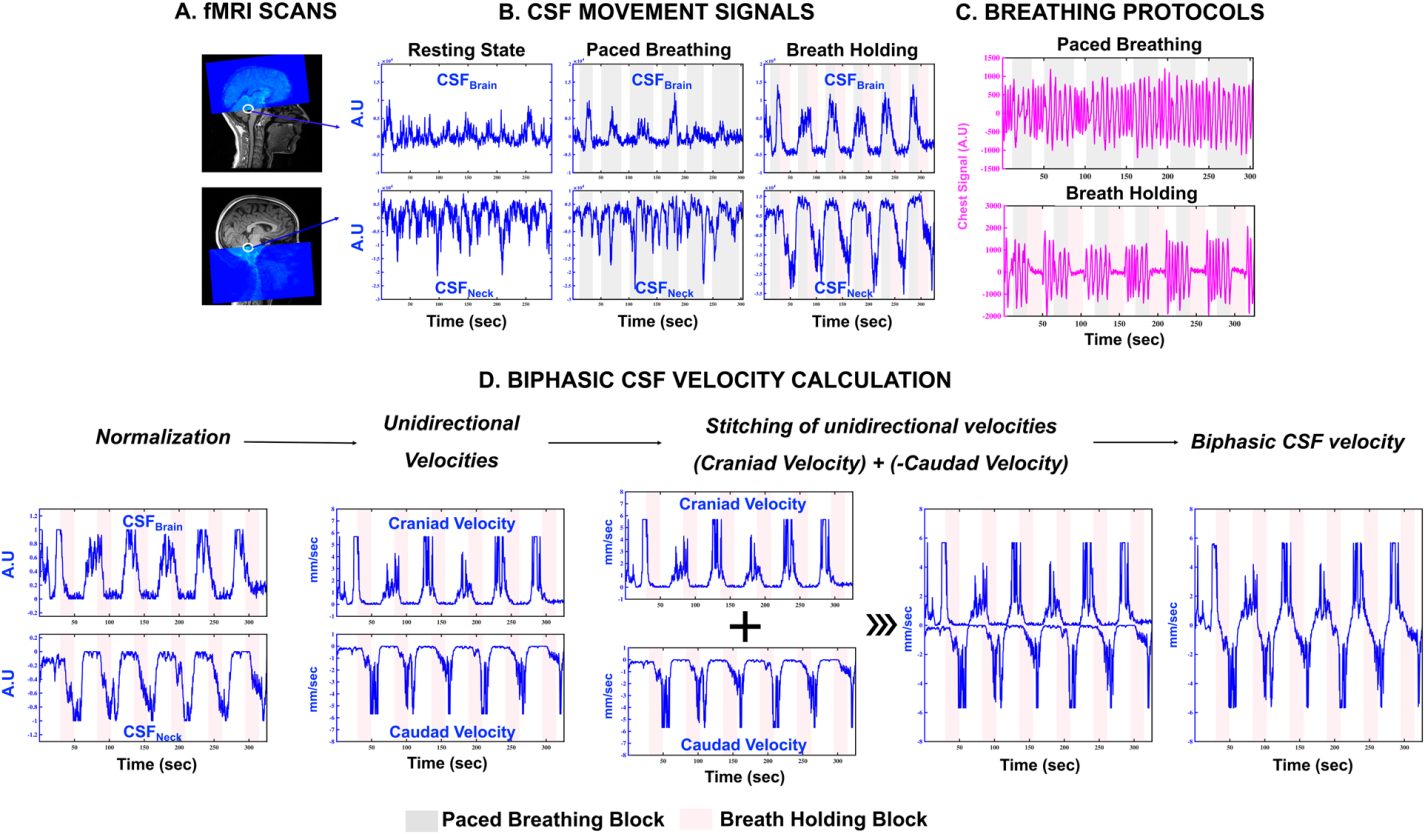
Overview of the experimental design and biphasic CSF velocity calculation: (A) Typical fMRI scan designs illustrating the capture of cranially directed CSF movement (CSF_Brain_) into the brain scan volume (top panel) and caudally directed CSF movement (CSF_Neck_) into the neck scan volume (bottom panel) utilizing inflow effect and (B) the corresponding CSF movement signals under resting state, paced breathing, and breath-holding conditions. (C) Breathing protocols and the corresponding chest belt respiratory signals. (D) Example of the generalized paradigm of biphasic CSF velocity calculation on breath holding. In panels B and D, CSF_Neck_and unidirectional caudad CSF velocity signals are inverted to reflect the caudad direction of movement. A.U, Arbitrary Units; CSF, Cerebrospinal Fluid; fMRI, functional Magnetic Resonance Imaging.

#### 2.2.1 Breathing challenges

The instructions for all breathing challenges were cued to the participants using a combination of textual and pictorial depictions compiled using PsychoPy (v2021.2.0) (Peirce et al., 2019), projected onto the mirror mounted on the MRI head coil. All participants also had a practice run of both challenges outside the scanner.

##### 2.2.1.1 Paced breathing challenge

The paced breathing challenge (Fig. 1C, top panel) used here consisted of six cycles of paced breathing at a frequency of 0.1667 Hz (i.e., 3-second inhale and 3-second exhale). The length of paced breathing in each cycle was between 18 and 48 seconds (3–8 repeats), followed by 15 seconds of normal breathing.

##### 2.2.1.2 Breath-holding challenge

The breath-holding challenge employed in this study was adapted from a paradigm widely used in previous fMRI studies (Bright & Murphy, 2013;H. S. Yang et al., 2020;Lipp et al., 2015). As depicted inFigure 1C(bottom panel), the challenge included six cycles of breath holding. Every cycle consisted of 18 seconds of paced breathing (three repeats of a 3-second inhale and 3-second exhale), 20 seconds of breath holding, and 15 seconds of normal breathing.

#### 2.2.2 MRI scans

All participants’ structural and functional MRI data were acquired using a 3T SIEMENS MRI scanner (Magnetom Prisma, Siemens Medical Solutions, Erlangen, Germany) with a 64-channel head-neck coil. The MR scans included structural T1-weighted MPRAGE (Magnetization Prepared Rapid Acquisition Gradient Echo – TR/TE: 2300/2.26 ms, 192 slices per slab, flip angle: 8°, resolution: 1.0 mm × 1.0 mm × 1.0 mm), resting state, and breathing challenge fMRI (FOV = 230 mm, acquisition matrix = 92 × 92, 48 slices, voxel size = 2.5 × 2.5 × 2.5 mm, TR/TE = 440/30.6 ms, echo-spacing = 0.51 ms, flip angle = 35°, multiband acceleration factor = 8, multi-slice mode: interleaved). Additionally, a chest belt was also worn by all participants to record respiration/chest signal (CS).

The fMRI scans employed here were carefully designed to capture the cranially and caudally directed CSF movements utilizing the inflow effect (Fultz et al., 2019;H. C. Yang et al., 2022) (seeFig. S1for an illustration in the Supplemental Material). As the CSF conduit between the brain and neck, we decided to assess the CSF movement at the level of the fourth ventricle. Additionally, the fourth ventricle’s narrow-tapered shape restricts CSF movement in other directions, enhancing the inflow effect. An intense CSF movement signal directed into the scan volume (i.e., Craniad CSF movement – CSF_Brain_and Caudad CSF movement – CSF_Neck_, respectively, for brain and neck scan volumes) can be captured by placing the bottom/top edge of the scan volume precisely at the fourth ventricle (seeFig. 1A). Accordingly, the slice of interest at the fourth ventricle was always acquired first in the scan.

#### 2.2.3 Data preprocessing

All MRI data were processed using FSL [FMRIB Expert Analysis Tool, v6.01; Oxford University, United Kingdom (Jenkinson et al., 2012)] and MATLAB (MATLAB 2020b; The MathWorks Inc., Natick, MA, 2000). The fMRI data were preprocessed in two different pipelines for extraction of CSF inflow signal and global signal (GS). In the first pipeline, only slice-timing (FSL SLICETIMER) correction was applied to the fMRI data before CSF signal extraction. The CSF inflow signal was then extracted from a suitable voxel at the center of the fourth ventricle (with negligible partial-volume effects from surrounding tissues) by overlaying the fMRI data over the structural T1-weighted image registered to the fMRI data. Motion correction was not applied in this pipeline since it distorts the slice position information and would be erroneous on edge slices where the tissue moves in and out of the imaging volume. Nevertheless, no significant correlations were detected between motion parameters (FSL MCFLIRT) and the CSF signals (Table S1in the Supplemental Material), ensuring they were not corrupted by motion. In the second preprocessing pipeline, motion correction (FSL MCFLIRT), slice-timing correction (FSL SLICETIMER), and spatial smoothing with a full width at half maximum (FWHM) of 5 mm isotropic Gaussian kernel were applied to fMRI data for subsequent analyses (Power et al., 2017). Further, the GS from across the entire brain was extracted from all resting state and breathing challenge brain scans after brain extraction.

#### 2.2.4 Data analysis

##### 2.2.4.1 Biphasic CSF velocity calculation

The basis for this calculation is the use of respiratory challenges as a physiological control condition, so as to bridge the independent CSF_Brain_and CSF_Neck_signals captured from separate brain and neck fMRI scans. Individuals’ unidirectional CSF velocities are computed from normalized fMRI data using the non-linear ‘Gao’ model outlined inDiorio et al. (2023), along with theoretical considerations fromGao et al. (1988). The model utilizes a plug flow assumption since the CSF flows at the center of the channel (i.e., fourth ventricle), is relatively uniform and less affected by the steep velocity gradients near the walls. Biphasic velocity temporal waveforms are computed by summing the velocity temporal waveforms with positive and negative representing craniad and caudad, respectively (seeFig. 1D).

##### 2.2.4.2 Amplitude variation calculation

The standard deviations of the biphasic CSF velocities were calculated to assess the variation in amplitude of these oscillations across the experimental conditions. For comparison, the biphasic CSF velocities and the corresponding standard deviations were also calculated during the resting state. Further, non-parametric Kruskal-Wallis tests were used to compute statistically significant differences in standard deviations between resting state and breathing challenges.

##### 2.2.4.3 Net CSF volume calculation

To quantify the net CSF volumes displaced during the breathing challenge conditions, the biphasic velocity temporal waveforms were first converted into corresponding biphasic CSF flow-rate temporal waveforms by multiplying with the area of the voxel (6.25 mm^2^). Further, the net CSF volume in each case was calculated by integrating the area under the flow-rate temporal series using MATLAB ‘trapz’. As a comparison, net CSF volume during the resting state was estimated as the difference between volumes integrated from unidirectional CSF flow-rate temporal waveforms. Further, statistical significance between the net CSF volumes displaced during each experimental condition was tested using non-parametric Kruskal-Wallis tests.

##### 2.2.4.4 Cross-correlation analysis

This analysis was performed to investigate the effects of paced breathing and breath-holding stimuli in controlling CSF movement at the level of the fourth ventricle. Specifically, these analyses were performed in two different frequency ranges as detailed below:

###### 2.2.4.4.1 Effects of LF cerebrovascular volume changes induced by respiratory challenges on CSF movement

Volume changes in the cerebrovasculature in response to fluctuations in the concentration of arterial CO_2_have been shown to occur in the LFO range (Birn et al., 2006;Wise et al., 2004). Therefore, the GS and CSF signals were linearly detrended, and bandpass filtered to the LFO range (0.01 Hz–0.1 Hz) before cross-correlation analysis. Further, maximum cross-correlation coefficients (MCCCs) and corresponding time delays were calculated (MATLAB ‘xcorr’, maximum lag range: ±15 seconds) between$\frac{d}{dt}$(GS) and biphasic CSF velocity signals for each participant in the LFO range for both experimental conditions. From among the computed CCCs, the MCCC was calculated as the absolute maximum value with its original arithmetic sign. In the LFO range, only those MCCCs that are greater than 0.3 or less than -0.3 are regarded as statistically significant (p-value < 0.01) (Hocke et al., 2016;Yao et al., 2019). Here, it is important to note that we use the derivative of the GS to effectively capture the cerebrovascular volume changes (H. C. Yang et al., 2022) in response to the breathing challenges. This is because the CSF moves in/out of the brain through the fourth ventricle only when there is a change in the cerebrovascular volume.

###### 2.2.4.4.2 Effects of intrathoracic pressure changes during paced breathing on CSF movement

To evaluate the effects of respiratory pressure changes during paced breathing, the temporal relationships between$\frac{d}{dt}$(CS) with GS and biphasic CSF velocity signals in the breathing frequency range for the paced breathing condition (0.1 Hz–0.2 Hz) were examined for each participant. All the signals were filtered into the paced breathing frequency range before the analysis. It must be noted here that the derivative of CS is employed in this analysis to reveal the instantaneous changes in the pressure differential between the right atrium and brain, during paced breathing challenge (Vijayakrishnan Nair et al., 2022). Based on previous research (Vijayakrishnan Nair et al., 2022), these calculations were performed by forcing the$\frac{d}{dt}$(CS) to lead the other signals assuming that respiration-related pressure change is the driver. Here, the MCCC’s were compared against the previously established threshold of ±0.26 for statistical significance (p-value < 0.05).

## 3 Results

### 3.1 Breathing challenges modulate CSF movement

**Fig. 2.**
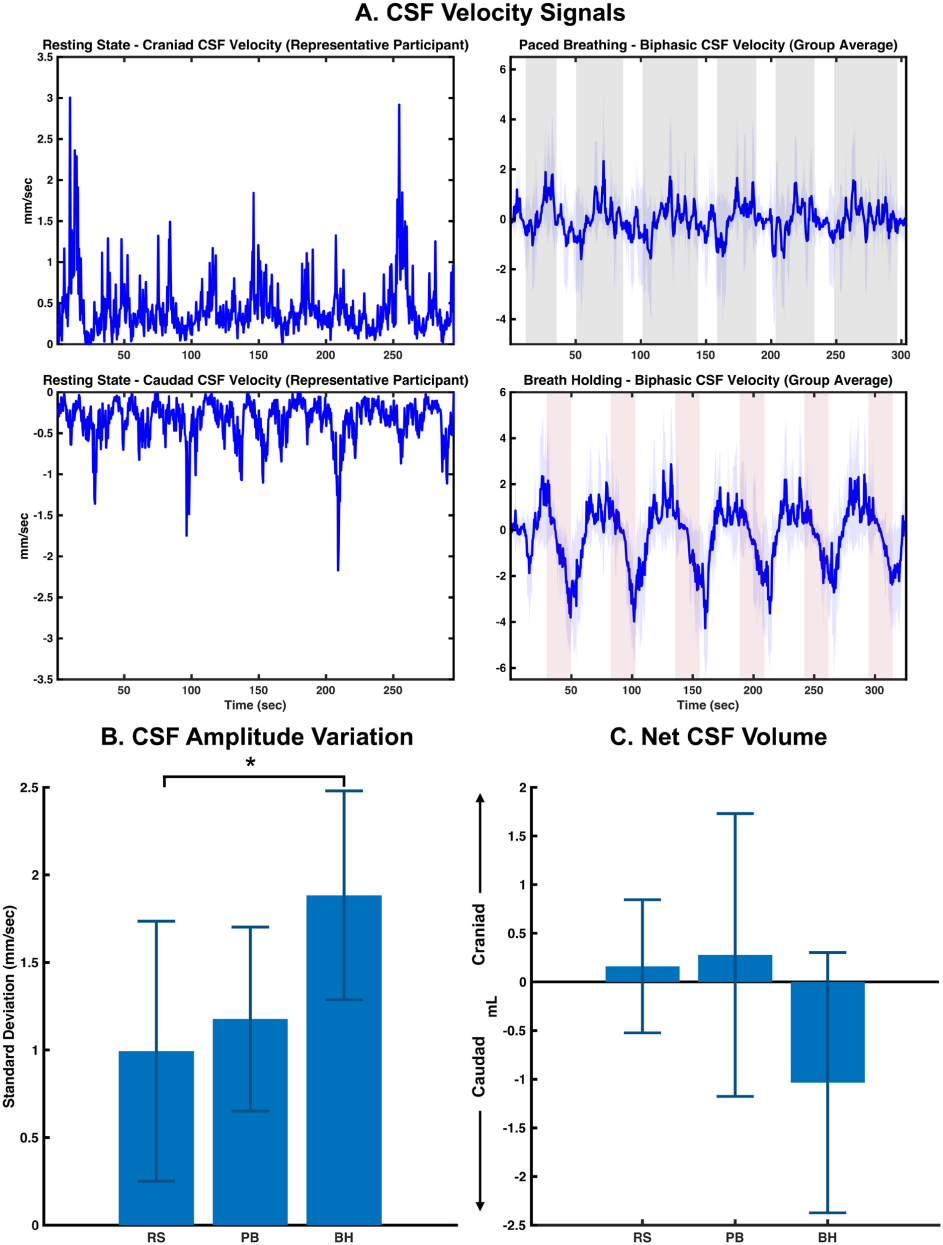
(A) Time series plots of unidirectional CSF velocity signals from a representative participant during resting state and group-averaged biphasic CSF velocity signals during breathing challenges. (B) Amplitude variation of biphasic CSF velocity signals quantified as standard deviations and (C) The net CSF volumes estimated from the velocities during all conditions. In panel A (breathing challenges), the mean signal for each case is illustrated with a thick line and the standard deviation across participants is represented by the shaded regions around the mean signal. Resting-state caudad CSF velocity signals are inverted and plotted to reflect the caudad direction of movement. CSF, Cerebrospinal Fluid; RS, Resting State; PB, Paced breathing; BH, Breath Holding; *, p-value <0.05.

Figure 2Adepicts the time series plots of unidirectional craniad and caudad CSF velocities from a representative participant (seeFig. S2in the Supplemental Material for resting-state velocities from all participants) in the resting state as well as the group-averaged biphasic CSF velocities during the paced breathing and breath-holding challenges. Here, it can be seen that the biphasic CSF velocity shows a net increase in the craniad direction during paced breathing blocks and a net increase in caudad direction during breath-holding blocks. More importantly, it can be seen that the amplitude of CSF velocity oscillations is much larger during the paced breathing and breath-holding challenges in comparison to resting state. In detail, the standard deviation (representing the amplitude variation) of biphasic CSF velocities during breath holding is significantly larger (p-value = 0.02, Cohen’s d = 1.32—very large effect size) than the resting state, whereas that of paced breathing is only relatively larger (p-value = 0.85, Cohen’s d = 0.28—small effect size) in comparison to resting-state conditions (seeFig. 2B). Furthermore, we also estimate that the breath-holding challenge generates a net volume of -1.04 ± 1.33 mL (p-value = 0.71, Cohen’s d = 1.10—very large effect size) displaced in the caudad direction, whereas paced breathing elicits a net volume of 0.28 ± 1.45 mL (p-value = 0.73, Cohen’s d = 0.25—small effect size) in the craniad direction, in comparison to a mere 0.16 ± 0.68 mL during resting state, across the entire duration of the corresponding scans (seeFig. 2C).

### 3.2 Effects of paced breathing on CSF dynamics

The results for paced breathing were separated into LFO and paced breathing frequency ranges, reflecting the two pathways of effects.

#### 3.2.1 Effects in the LFO range

**Fig. 3.**
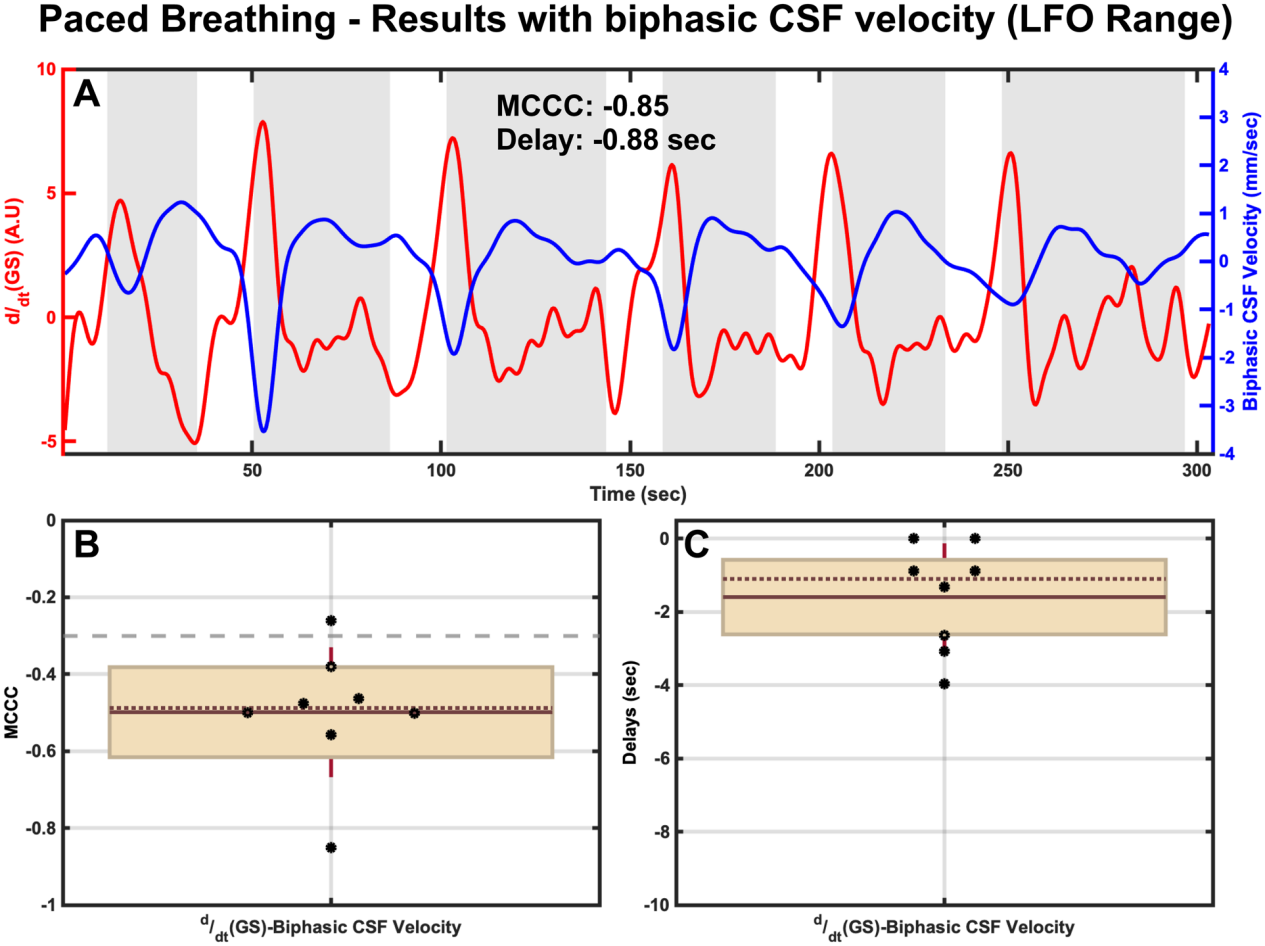
Results of MCCCs and corresponding delays during paced breathing between (A)$\frac{\text{d}}{\text{dt}}$(GS) and reconstructed biphasic CSF velocity for a representative participant with corresponding group results of (B) MCCCs and (C) Delays. In panels B and C, the brown horizontal solid line and dotted line respectively represent the mean and the median, the brick red whiskers represent one standard deviation of the data points jittered over a 95 percent confidence interval in cream, and the gray dashed line (panel B) represents the threshold of statistical significance for MCCCs in the LFO range. CSF, Cerebrospinal Fluid; GS, Global Signal; A.U, Arbitrary Units; MCCC, Maximum Cross-Correlation Coefficients; LFO, Low Frequency Oscillations (0.01 Hz–0.1 Hz).

MCCCs and corresponding delays between$\frac{d}{dt}$(GS) and reconstructed biphasic CSF velocity in the LFO range for a representative participant can be found inFigure 3A(paced breathing). The signals clearly depict that the change in GS (i.e., a gradual decrease—captured using$\frac{d}{dt}$(GS)) is followed shortly by a clear upward trend (i.e., indicative of the increasing craniad CSF_Brain_) in reconstructed biphasic CSF velocity signal during the paced breathing periods. Looking at the cross-correlation results for this participant, it can be seen that the$\frac{d}{dt}$(GS) exhibits a significant negative correlation with reconstructed biphasic CSF velocity (MCCC < -0.3) with a delay of -0.88 seconds. Here, negative delay indicates that the$\frac{d}{dt}$(GS) leads the CSF movement signals. Group results (Fig. 3Band3C) further confirm these findings with mean correlations of -0.50 ± 0.17 between$\frac{d}{dt}$(GS) and biphasic CSF velocity at mean time delays of -1.60 ± 1.47 seconds. Correlations and delays between$\frac{d}{dt}$(GS) and unidirectional CSF signals (CSF_Brain_and CSF_Neck_) in the LFO range also show similar results (seeFig. S3in the Supplemental Material).

#### 3.2.2 Effects in the paced breathing frequency range

Group-averaged time series plots of reconstructed biphasic CSF velocity with$\frac{d}{dt}$(CS) and with GS in the paced breathing frequency range can be found inFigure 4Aand4Brespectively. A magnified version of these signals within a single PB block is also illustrated inFigure 4Cand4D. The temporal patterns of these signals clearly demonstrate that positive changes in the$\frac{d}{dt}$(CS) (representing inspiration) leads to a decrease in GS and is followed by an increasing upward trend in biphasic CSF velocity (indicative of craniad movement). On the other hand, negative changes in the$\frac{d}{dt}$(CS) (representing expiration) lead to an increase in GS and are followed by an increasing downward trend in biphasic CSF velocity (indicative of caudad movement). Cross-correlations to quantify these relations were only calculated with the unidirectional CSF signals (CSF_Brain_and CSF_Neck_), since the CS representing the intrathoracic volume/pressure were acquired separately (seeFig. S3in the Supplemental Material).

**Fig. 4.**
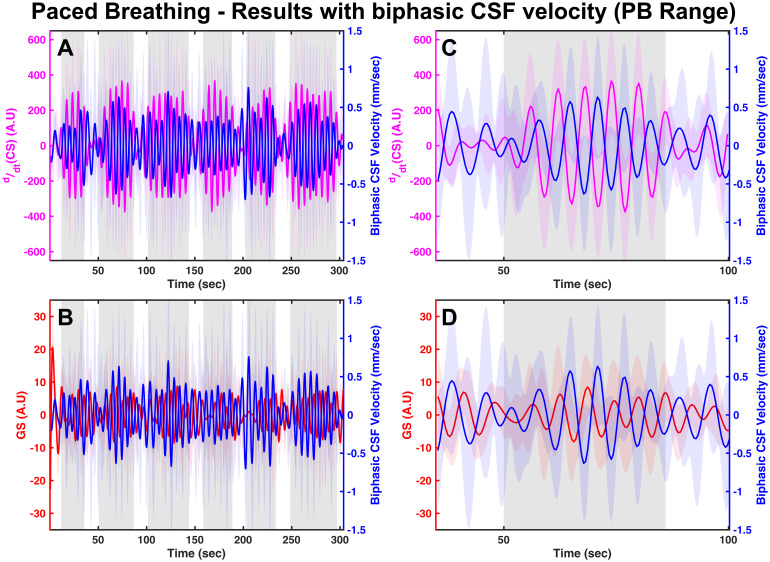
Group-averaged time series plots of reconstructed biphasic CSF velocity with (A)$\frac{\text{d}}{\text{dt}}$(CS) and (B) GS in the paced breathing frequency range (0.1 Hz–0.2 Hz). A magnified version of the plots in (A and B) within a single PB block can be found in respectively in (C and D). In all panels, the mean signal for each case is illustrated with a thick line and the standard deviation across participants is represented by the shaded regions around the mean signal. CSF, Cerebrospinal Fluid; CS, Chest Signal; GS, Global Signal; A.U, Arbitrary Units; PB, Paced breathing.

### 3.3 Effects of breath holding on CSF dynamics

**Fig. 5.**
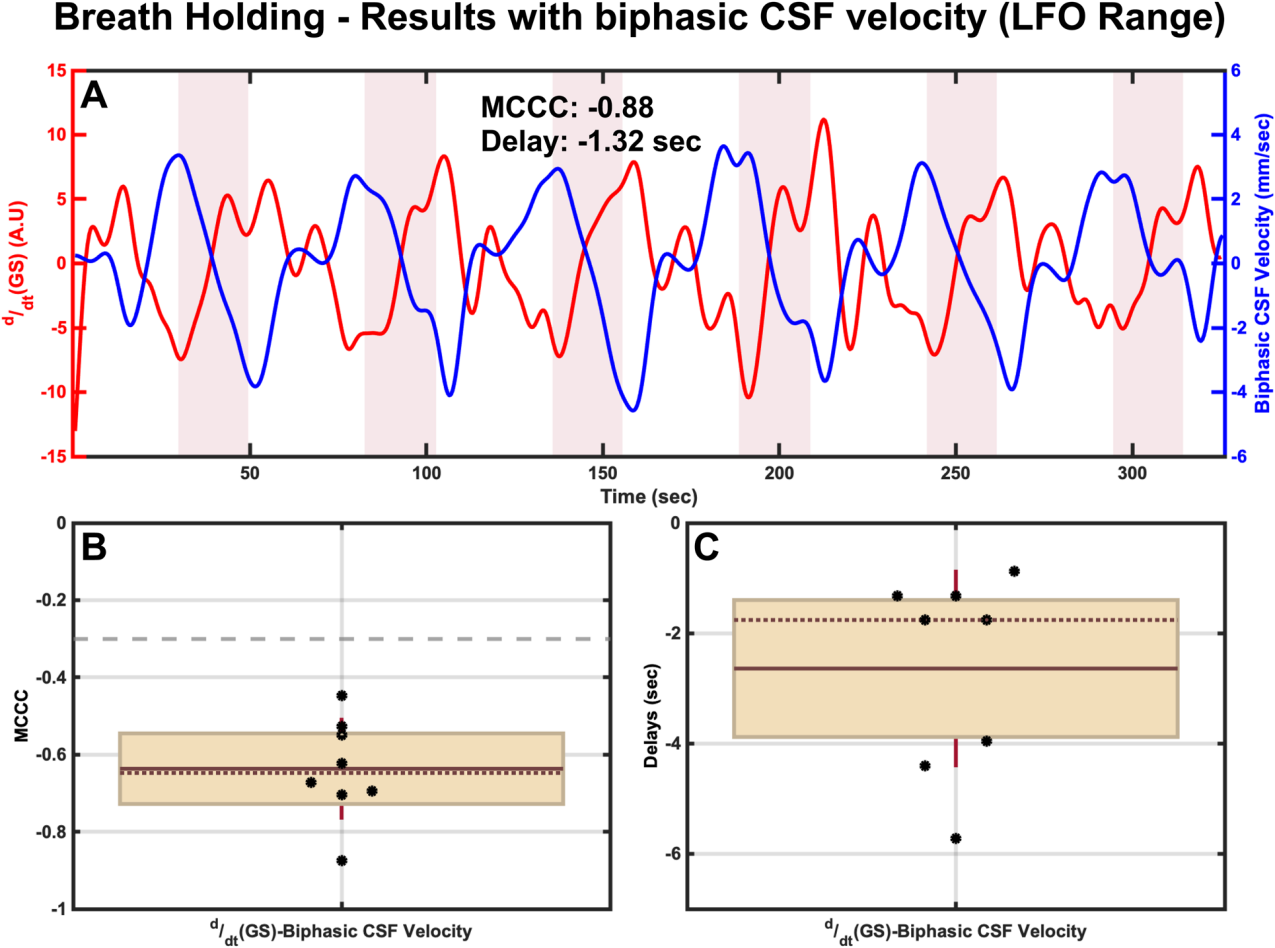
Results of MCCCs and corresponding delays during breath holding between (A)$\frac{\text{d}}{\text{dt}}$(GS) and reconstructed biphasic CSF velocity for a representative participant with corresponding group results of (B) MCCCs and (C) Delays. In panels B and C, the brown horizontal solid line and dotted line respectively represent the mean and the median, the brick red whiskers represent one standard deviation of the data points jittered over a 95 percent confidence interval in cream, and the gray dashed line (panel B) represents the threshold of statistical significance for MCCCs in the LFO range. CSF, Cerebrospinal Fluid; GS, Global Signal; A.U, Arbitrary Units; MCCC, Maximum Cross-Correlation Coefficients; LFO, Low Frequency Oscillations (0.01 Hz–0.1 Hz).

MCCCs and corresponding delays between$\frac{d}{dt}$(GS) and reconstructed biphasic CSF velocity in the LFO range for a representative participant are illustrated inFigure 5A(breath holding). The signals clearly depict the change in GS (i.e., a gradual increase—captured using$\frac{d}{dt}$(GS)), which is followed shortly by a clear downward trend (i.e., indicative of the increasing caudad CSF_Neck_) in the reconstructed biphasic CSF velocity signal during the breath-holding periods. Looking at the cross-correlation results for this representative participant, it can be seen that the$\frac{d}{dt}$(GS) exhibits a significant negative correlation with reconstructed biphasic CSF velocity (MCCC < -0.3) at a time delay of -1.32 seconds. Here, negative delay indicates that the$\frac{d}{dt}$(GS) leads the CSF movement signals. Group results (Fig. 5Band5C) further confirm these findings with mean correlations of -0.64 ± 0.13 between$\frac{d}{dt}$(GS) and biphasic CSF velocity with the$\frac{d}{dt}$(GS) signal leading the biphasic CSF velocity signal by average time delays of -2.64 ± 1.79 seconds. Correlations and delays between$\frac{d}{dt}$(GS) and unidirectional CSF signals (CSF_Brain_and CSF_Neck_) in the LFO range also show similar results (seeFig. S3in the Supplemental Material).

## 4 Discussion

Results from the current study are three-fold—First and most importantly, our data reveal that both respiratory challenges, paced breathing and breath holding, can modulate the magnitude and direction of CSF movement at the level of the fourth ventricle, based on the cerebral blood volume (CBV)–CSF model (H. C. Yang et al., 2022). Second, we report, for the first time, a novel methodology that allows us to compare biphasic CSF movements (in the fourth ventricle) from independently captured direction-sensitive CSF inflow signals using the respiratory challenges as a physiological control condition. Third, using this method, we demonstrate that the breathing challenges, particularly breath holding, elicit much larger CSF flow dynamics (in both directions) compared to the resting-state conditions. However, we did not detect any significant overall flows during these challenges. Below, we discuss the implications of these results.

### 4.1 CO _2_ /LFO pathways of CSF movement modulation

Our data during paced breathing sessions explicitly illustrate hypocapnia as the prominent effect of this challenge, which controls CSF movement via the CO_2_/LFO pathway (SeeFig. 6A(green panel) for a pictorial summary of this pathway). In detail, paced breathing challenge is known to result in an increased tidal ventilatory volume (i.e., hyperventilation) and, thereby, elicit hypocapnia (seeFig. S4in the Supplemental Material), a condition characterized by the reduction of arterial partial pressure of CO_2_. In turn, this results in a rise in intracellular pH, which further induces evident vasoconstriction (Ogoh, 2019;Yoon et al., 2012) and a consequent rapid decrease in CBV (Bright et al., 2009;Posse et al., 1997). This rapid decrease in CBV can be seen during each paced breathing period (Fig. 3a) as a sharp drop in the$\frac{d}{dt}$(GS) (i.e., CBV drops even faster), which then leads to an increase in craniad CSF movement to compensate for this decreased CBV, as per the Monro-Kellie principle (Mokri, 2001). This negative change in$\frac{d}{dt}$(GS), and the consequent net increase in CSF velocity in the craniad direction, with an average delay of 1.60 ± 1.47 seconds can be clearly seen from our data during the paced breathing challenge (seeFig. 3andFig. S3in the Supplemental Material). Lastly, during the intermittent normal breathing periods, the hypocapnia ends and the CBV increases back to normal that leads to the increase in$\frac{d}{dt}$(GS) signal and in caudal CSF movement. In alignment with these findings, existing research also indicates that CSF moves craniad approximately 2 to 3 seconds after a decrease in the global signal during periods of deep breathing (Picchioni et al., 2022;Wang et al., 2022).

**Fig. 6.**
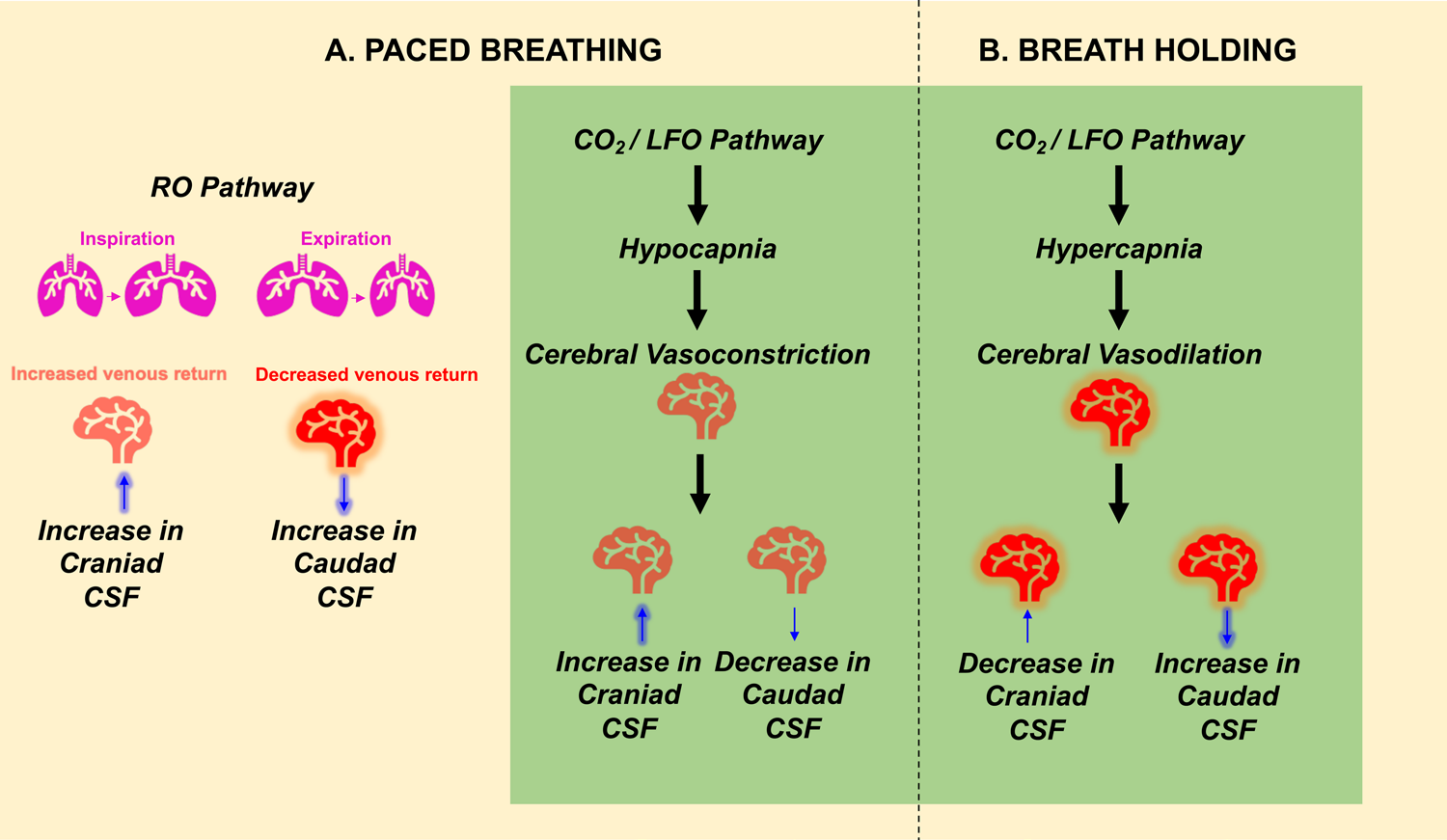
Summary of physiological mechanisms/pathways by which respiratory challenges—(A) paced breathing and (B) breath-holding control CSF dynamics. Note that paced breathing exerts its effects through two pathways across different frequency ranges—RO pathway and CO_2_/LFO pathway, whereas breath holding elicits its influence through the CO_2_/LFO pathway only. CSF, Cerebrospinal Fluid; LFO, Low Frequency Oscillations (0.01 Hz–0.1 Hz); RO, Respiratory Oscillations (0.1 Hz–0.2 Hz).

On the other hand, our data from the breath-holding paradigm show that this challenge elicits a positive change in$\frac{d}{dt}$(GS), and is shortly followed by a net increase in CSF velocity in the caudad direction, about 2.64 ± 1.79 seconds later (seeFig. 5andFig. S3in the Supplemental Material). This opposite effect can be explained by hypercapnia which is well known to occur during breath-holding challenges (Bright & Murphy, 2013;Larsen et al., 2021;Magon et al., 2009;Raitamaa et al., 2019;Thomason et al., 2005). This condition, characterized by the elevated levels of blood CO_2_, in turn, leads to significant cerebral vasodilation through the reduction of intracellular pH and consequent relaxation of smooth muscle cells in the arteries, as well as through enhanced secretion of relaxation-promoting factors from the vascular endothelium (Ogoh, 2019;Yoon et al., 2012). This potent vasodilation induced by hypercapnia leads to an increase in cerebral blood volume. This, in turn, leads to an increase in caudad CSF movement to compensate for the increase in cerebral blood volume during the breath hold induced hypercapnia phase (seeFig. 6B(green panel) for a pictorial summary of this pathway). Lastly, during the intermittent normal breathing periods (similar to that of paced breathing challenge, while in the opposite direction), the hypercapnia ends and the CBV decreases back to normal that leads to the decrease in$\frac{d}{dt}$(GS) signal and increase in craniad CSF movement (Fig. 5A).

In addition to these direction-specific modulation of CSF movements in the LFO range, our data also show that these breathing challenges elevate the amplitude variation in CSF movement oscillations at the fourth ventricle, especially in the LFO range (seeFig. 2Aand2B). In detail, we found that the mean amplitude fluctuations of biphasic CSF velocities during paced breathing/breath-holding challenges respectively are relatively/significantly larger than the resting-state conditions (seeFig. 2B). Furthermore, we also found that that the amplitude fluctuations in CSF movements induced by breathing challenges are comparable to those observed during NREM (Non Rapid Eye Movement) sleep. Specifically, comparing the current results with an analysis with the NREM sleep data published previously by our group (Vijayakrishnan Nair et al., 2023), we show that the amplitude variations during breath holding are, in fact, relatively larger than that observed during light NREM sleep (seeFig. S5in the Supplemental Material).

These findings from our study offer compelling insights for several reasons. First, the application of our CBV-CSF coupling model demonstrates the feasibility of employing specific methods, notably respiratory challenges, to modulate the low-frequency CSF flow in the fourth ventricle through alterations in CBV. Disruptions in these oscillations, as shown byKim et al. (2020), may impede perivascular CSF flow (Kim et al., 2020) and contribute to the progression of Alzheimer’s disease (Di Marco et al., 2015). Our study demonstrates a method for actively modulating low-frequency CBV through respiratory challenges, significantly increasing CSF dynamics in the fourth ventricle compared to the resting state. Second, by implementing a novel biphasic method, we have identified that the CSF flow is induced bidirectionally, with both directions achieving comparable volumes. This results in a minimal net flow during the experimental period, of ~5 minutes (discussed inSection 4.4). However, the relationship between enhanced low-frequency CSF flow dynamics (in both directions) and their effects on the interactions between CSF and ISF, as well as the overall impact on brain clearance, remains ambiguous. There exists a significant knowledge gap in connecting our observations at the fourth ventricle on a macroscale with the microscale processes involved in brain clearance. A recent study employing tracer-enabled two-photon imaging in live mice revealed that the perivascular CSF spaces in the brain exhibit slow, yet substantial, bidirectional low-frequency oscillations. These oscillations correlate with changes in the diameters of pial arteries and penetrating arterioles during NREM sleep (Bojarskaite et al., 2023), a period when the clearance of brain metabolites has also been shown to be maximized (Xie et al., 2013). These findings suggest that the bidirectional movements in CSF dynamics could potentially enhance the exchange between CSF and ISF downstream, thereby improving clearance. Nonetheless, this hypothesis requires empirical validation. Our methodology promises clinical relevance, contingent upon the verification of this theory.

### 4.2 Intrathoracic pressure effects on CSF movement

The control of CSF movement by intrathoracic pressure changes during respiration has been validated both during resting state (Friese et al., 2004;Klose et al., 2000;Vijayakrishnan Nair et al., 2022) and paced breathing conditions (Chen et al., 2015;Dreha-Kulaczewski et al., 2015,^2017^;Yamada et al., 2013). Our results in the paced breathing frequency range (seeFig. 4) also validate this pathway (seeFig. 6A(yellow panel) for a pictorial summary). Similar to the resting state (Vijayakrishnan Nair et al., 2022), our results show that the paced breathing also affects the cerebral blood volume oscillations in about a second, and then the CSF movement oscillations another second later (seeFig. S3in the Supplemental Material). However, we did not find any large CSF movements in the paced breathing frequency range compared to the CO_2_/LFO effects. In summary, the paced breathing protocol enhances CSF flow dynamics in both directions through two different mechanisms: (1) The modulation of intrathoracic pressure, which occurs with each breath, and (2) the alteration of cerebrovascular tone through induction of hypocapnia during periods of paced breathing which occurs more slowly and exerts a more substantial effect (seeSection 4.3for a quantitative comparison).

### 4.3 CO _2_ /LFO effects versus intrathoracic pressure effects

We also performed a power analysis of the CSF velocity estimates (seeFig. S6in the Supplemental Material) during the breathing challenges to quantify the relative contributions from LFOs and intrathoracic thoracic pressure changes. This shows that about 71.52 ± 9.02% of the observed CSF motion during breath holding comes from the LFO range, implying that the changes in concentration of arterial CO_2_(i.e., hypercapnia) were the primary contributing factor for this large caudad CSF movement. On the other hand, in the case of paced breathing, only 24.01 ± 10.39% of the CSF motion lies in the paced breathing frequency range despite an increment in the strength of the thoracic pump, whereas about 53.12 ± 15.27% comes from the LFO range, suggesting that CO_2_concentration change (i.e., hypocapnia) associated with paced breathing is the major contributor to the observed CSF movement. Contributions from cardiac frequencies on average were small during both challenges (8.95 ± 4.83% during paced breathing and 7.98 ± 5.73% during breath holding). Similar results can be found from power analysis of unidirectional CSF inflow signals as well during both challenges (seeFig. S6in the Supplemental Material). These findings thereby underline the capability of CO_2_as a vasoactive stimulus to bring about changes in LF cerebral hemodynamic oscillations and CSF movement.

### 4.4 Net CSF volume flows

The new methods used in this study allowed us to quantitatively assess the CSF volume estimates from the biphasic velocities during the breathing challenges. As highlighted inSection 4.1, during both the respiratory challenge and normal breathing periods, the inflow and outflow of CSF are relatively balanced, resulting in a minimal net flow throughout the entirety of the experiment. Nevertheless, a net volume of CSF moving towards the spinal canal compared to the resting state (Cohen’s d = 1.10—very large effect size) was detected for a total duration of ~5 minutes during breath holding. As shown inFigure 5, the CSF moves in both directions during each epoch of breath holding. However, our results indicate that caudad CSF flow is bigger than craniad. Accumulative effects of 6 epochs led to about 1.04 mL net flow into the spinal canal. This implies that even as the brain volume (i.e., cerebral blood volume) oscillates during each breath-holding epoch, it also “swells” slowly (over 5 minutes) due to the mild build-up of the residual CO_2_(hypercapnia) over the periods of the breath-holding challenge. In contrast to breath holding, the net CSF volume displaced in the craniad direction by paced breathing on average was only slightly larger (Cohen’s d = 0.25—small effect size) with high variability across participants, compared to the resting state (seeFig. 2C). This may be attributed to the fact that hypocapnia induced by the paced breathing paradigm employed here is only short-lived and mild (Bright et al., 2009). Moreover, this might also be related to the higher inter-individual variability in cerebrovascular responses to paced breathing tasks (Birn et al., 2008;Sousa et al., 2014), which is observed in our data as well. Despite the higher variability in the net CSF volume, the average CSF responses during the individual paced breathing blocks always showed a craniad orientation (seeFig. 2Aand2B). Using the same physiological framework, this phenomenon may be attributed to gradual brain “shrinkage” over a period of approximately 5 minutes, likely resulting from the slow reduction in CO_2_levels (hypocapnia) during the paced-breathing periods.

The net flow rate of CSF through the fourth ventricle is estimated to be in the range of 0.2–0.6 mL/min (Lindstrøm et al., 2018;Piechnik et al., 2008), presenting challenges for detection via MRI over short durations, particularly with fMRI that depends on the inflow effect in a singular direction. In this study, we demonstrated that our innovative method can identify such slow flows during respiratory challenges. Further refinements are necessary for wider application. However, both large bi-directional CSF flow dynamics and net CSF flows have been implicated in glymphatic function through animal model studies (Bojarskaite et al., 2023;Xie et al., 2013), albeit their precise contributions remain to be fully elucidated.

## 5 Limitations and Conclusions

The biphasic approach utilized in this study is an approximation. The acquisition of the two scans at distinct time points could potentially introduce challenges. Moreover, the velocity quantification model employed here cannot estimate velocities greater than a ‘critical value’ which is dependent on the fMRI voxel size and temporal resolution.Wang et al. (2022)also estimated CSF flow velocities in the fourth ventricle by aligning measured CSF tags with a simulated flow dictionary across various frequencies. However, their method, based on a limited set of velocities over multiple cardiac cycles, may bias slow velocity estimates (Wang et al., 2022). Conversely, our implementation (Diorio et al., 2023) uses a more direct conversion technique, but further refinement and validation are necessary for velocity estimations from inflow effect. Finally, it must be noted that the CSF flowrate and net volume calculations performed in this study did not consider the complex geometry/cross-sectional area of the fourth ventricle, since the inflow signals arise from a single voxel at the center of the fourth ventricle. Nonetheless, the assessment of net CSF volume flows remains valid even without reliance on the velocity methodology (seeFig. S7in the Supplemental Material, where we conducted similar calculations without velocity/flowrate estimations). In conclusion, our data demonstrate the potential of simple respiratory challenges such as paced breathing and breath holding to alter cerebrovascular oscillations (potentially through blood CO_2_changes) and thereby in modulating directed CSF movement at the level of the fourth ventricle. Moreover, these breathing challenges also elicit much larger CSF movement fluctuations comparable to that of NREM sleep. Lastly, we also report on a novel method to calculate and quantify biphasic CSF movement in the fourth ventricle utilizing the breathing challenges as a physiological control condition.

## Supplementary Material

Supplementary Material

## Data Availability

All data and code used in this manuscript will be available upon reasonable request due to privacy/ethical restrictions.
