# Supplementary material for: Using respiratory challenges to modulate CSF movement across different physiological pathways: An fMRI study
Source: Imaging Neurosci (Camb). 2024 Jun 10;2:imag-2-00192. doi: 10.1162/imag_a_00192 (PMC12272236; doi:10.1162/imag_a_00192)
Supplement: Supplementary Material [file imag_a_00192-supp.pdf]

Supplemental Material

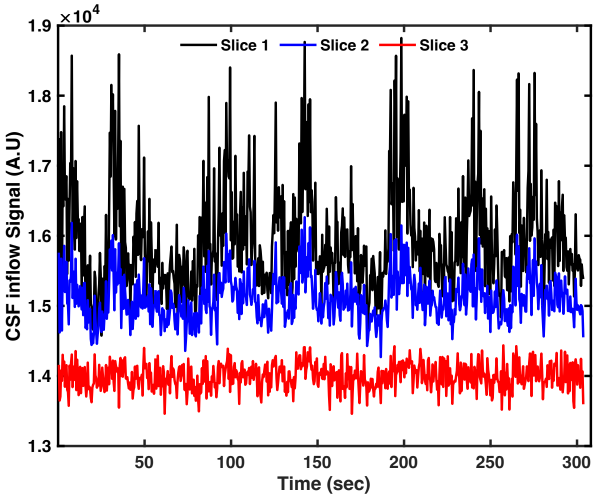

Figure S1: Illustration of fMRI inflow effect. it can be seen that an intense single-voxel CSF movement signal is captured from the edge slice (i.e., slice 1 in the figure), arising from inflow effect. At the same time, the intensity of similar single-voxel signals from subsequent inner slices (i.e., slices 2 and 3) is much lower due to decreased sensitivity of these slices to inflow effect. This is because, the fluid particles have to move further up into the scan volume to reach these slices and (has already encountered the RF pulses) therefore induce a signal of lower intensity. These results confirm that the single-voxel CSF movement signals captured from the edge slices are predominantly originating from inflow effect. Moreover, this also show that averaging of these signals or extracting a signal from across the fourth ventricle region would potentially reduce the signal to noise ratio.

	Resting State Brain Scans						Paced Breathing Brain Scans						Breath Holding Brain Scans					
Parameter / Participant	r_x	r_y	r_z	t_x	t_y	t_z	r_x	r_y	r_z	t_x	t_y	t_z	r_x	r_y	r_z	t_x	t_y	t_z
1	0	-0.03	0.08	0.2	-0.02	-0.07	-0.1	0.01	0.14	0.19	-0.28	-0.02	0	0.2	0	-0.01	0.07	-0.17
2	0.21	0.02	-0.01	0	-0.1	-0.08	-0.17	-0.08	0.17	-0.23	-0.26	0.07	-0.05	-0.08	0.3	-0.35	-0.09	0.08
3	0.12	-0.01	-0.03	-0.01	-0.04	-0.26	-0.11	0.24	0.09	0.09	-0.25	0.21	-0.12	0.08	-0.08	-0.08	0.02	0.3
4	0.01	-0.01	0.02	0.06	-0.26	-0.02	-0.15	0.09	-0.12	-0.05	-0.08	-0.01	0.02	0.07	0.01	0.19	0.12	0.21
5	-0.07	-0.02	0	0.01	-0.14	0.01	-0.06	-0.01	0.08	0.04	-0.28	0.23	-0.03	-0.07	-0.05	0.02	-0.1	0.07
6	0.05	0.12	0.12	0.06	0	-0.07	0.09	0.18	-0.02	-0.02	-0.01	-0.1	-0.09	0	-0.02	-0.02	-0.08	-0.05
7	-0.09	0.07	-0.05	-0.1	0.02	0.08	-0.01	0.01	-0.02	-0.01	0.03	-0.05	0.08	-0.1	0.28	0.11	0.11	0
8	-0.1	0.05	-0.04	-0.13	0	-0.12	0.02	0.02	-0.02	-0.01	0.04	-0.08	0.02	-0.04	0	-0.01	0.05	-0.07
Mean	0.02	0.02	0.01	0.01	-0.07	-0.06	-0.06	0.06	0.04	0	-0.14	0.03	-0.02	0.01	0.06	-0.02	0.01	0.05
Standard Deviation	0.11	0.05	0.06	0.1	0.09	0.1	0.09	0.11	0.1	0.12	0.15	0.13	0.07	0.1	0.15	0.16	0.09	0.15
	Resting State Neck Scans						Paced Breathing Neck Scans						Breath Holding Neck Scans					
Parameter / Participant	r_x	r_y	r_z	t_x	t_y	t_z	r_x	r_y	r_z	t_x	t_y	t_z	r_x	r_y	r_z	t_x	t_y	t_z
1	0.02	0.16	-0.19	-0.07	0.02	0.08	0.06	-0.09	0.03	-0.03	-0.03	-0.06	-0.19	-0.19	0.08	-0.13	0.13	0.03
2	-0.04	0.06	-0.16	-0.1	0.02	0.07	0.29	0.14	-0.22	0.26	0.03	-0.04	0.1	-0.05	-0.19	0.15	0.02	-0.15
3																		
4	0.07	0.01	-0.02	0.17	-0.09	-0.11	0.15	-0.05	0.02	0.02	-0.11	0.01	0.17	0.18	-0.08	-0.06	-0.06	-0.05
5	0.21	0.01	0	0	-0.18	-0.34	0.34	0.09	-0.01	0.15	-0.25	-0.2	0.2	0.25	0.19	0.31	-0.36	-0.12
6	0.03	-0.05	0.05	-0.02	-0.03	0.05	0	-0.04	0	-0.1	0	0.02	0.08	0.01	-0.03	-0.04	-0.07	0.02
7	-0.33	0	0	-0.09	0.14	-0.09	-0.33	-0.4	0.23	-0.14	-0.08	-0.2	-0.12	-0.08	0.07	-0.05	-0.02	0.04
8	-0.18	0.03	-0.03	0.03	0.2	0.12	-0.11	0.16	-0.1	0.05	-0.03	0.08	0.08	-0.04	0.08	0.09	0.01	-0.22
Mean	-0.03	0.03	-0.05	-0.01	0.01	-0.03	0.05	-0.03	-0.01	0.03	-0.06	-0.05	0.03	0.02	0.02	0.04	-0.04	-0.08
Standard Deviation	0.18	0.07	0.09	0.1	0.13	0.16	0.22	0.18	0.13	0.13	0.09	0.1	0.14	0.14	0.12	0.14	0.14	0.11

Table S1: Correlations between motion parameters (FSL MCFLIRT) and caudally directed CSF signals for each participant. From the results, it can be seen that there exist no noteworthy correlations between motion parameters and the CSF signals. This confirms that motion did not affect the CSF signals extracted from the fMRI scans. $r_{(x\text{-}z)}$: MCFLIRT estimated rotations; $t_{(x\text{-}z)}$: MCFLIRT estimated translations.

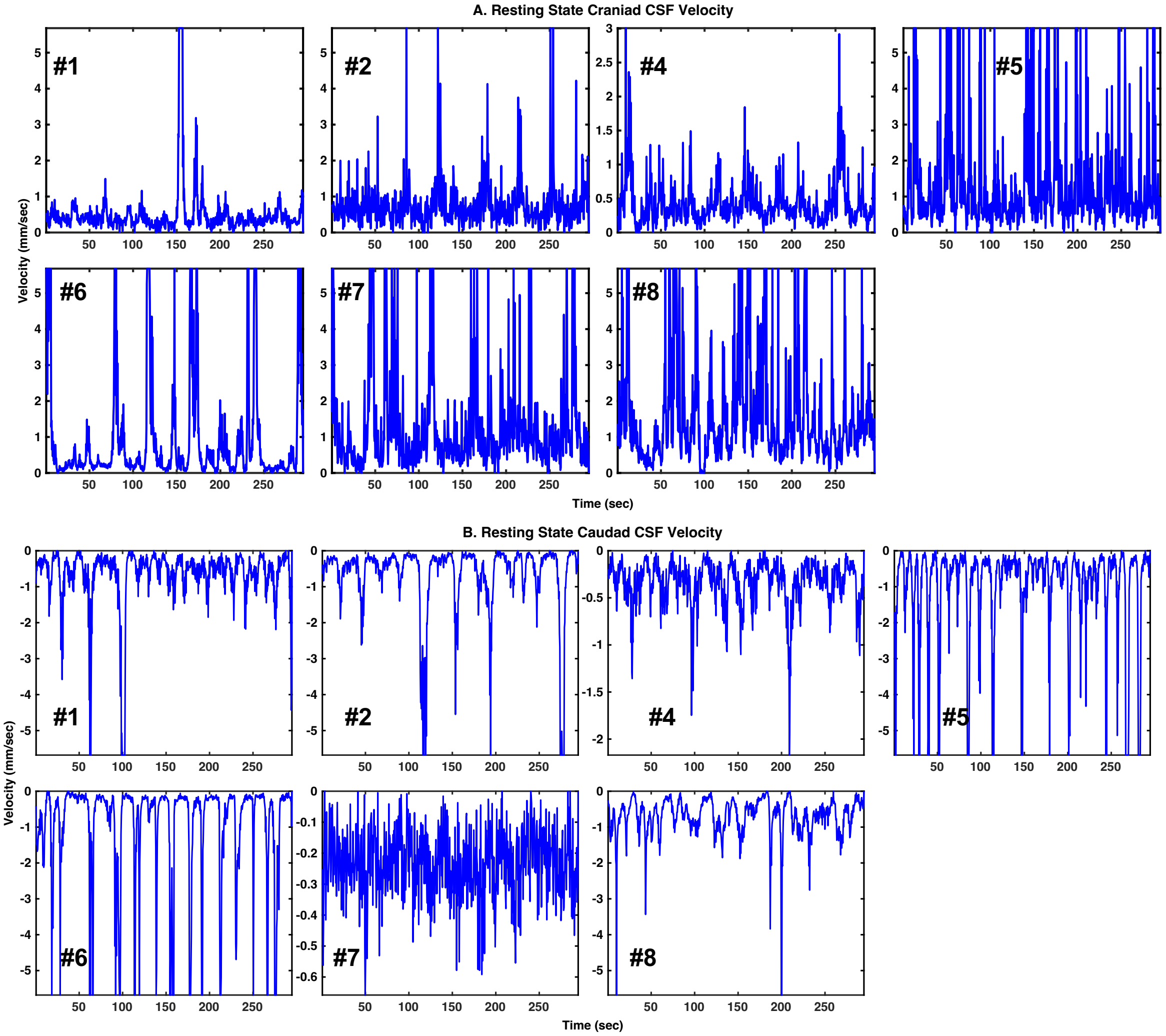

Figure S2: Time series plots of unidirectional CSF velocity signals from all participants during resting state in (A) craniad and (B) caudad directions. Resting state caudad CSF velocity signals are inverted and plotted to reflect the caudad direction of movement. Note that participant #3 is excluded, since the resting state neck scan for this participant was not accurately acquired. CSF – Cerebrospinal Fluid.

Results with Unidirectional CSF movement time series

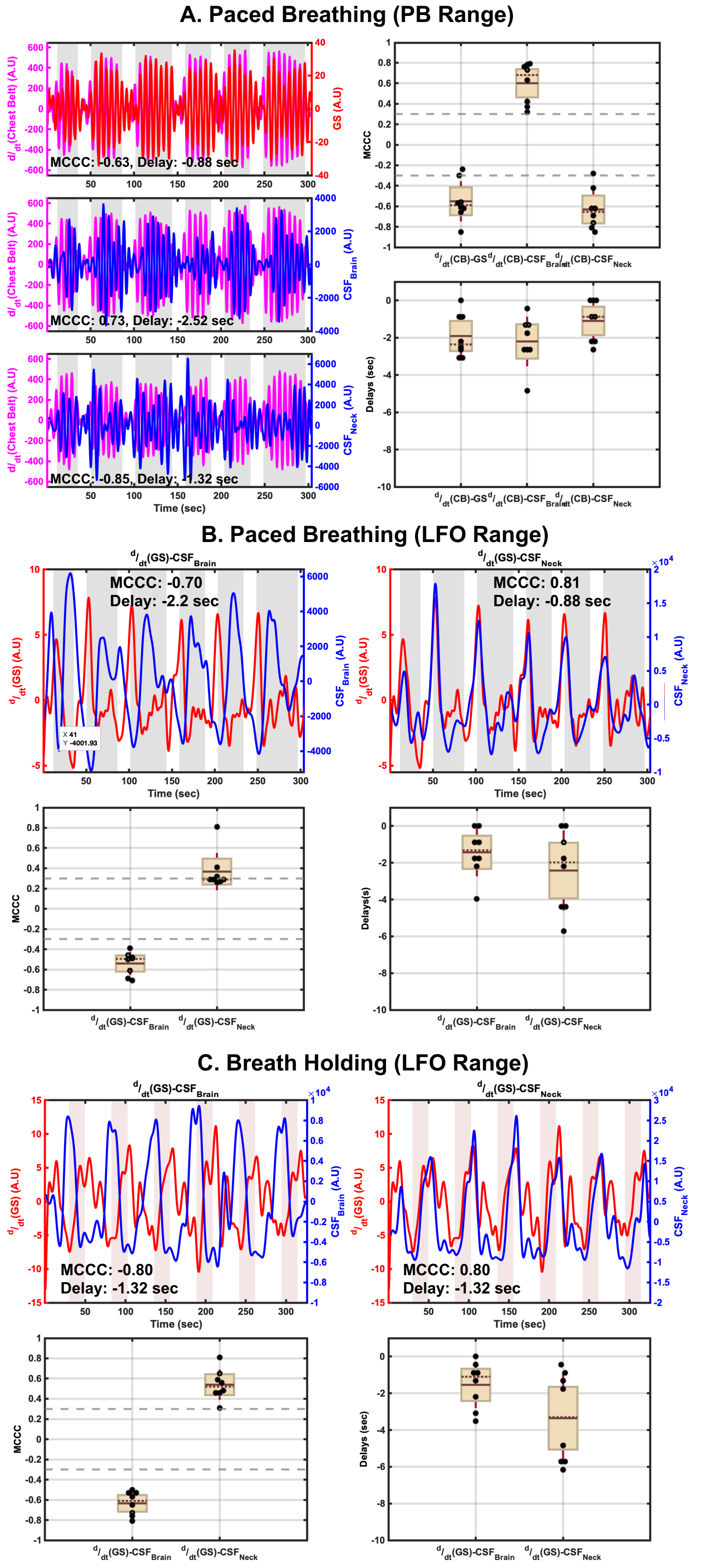

Figure S3: Cross-Correlations and delays between changes in global brain/chest oscillations and unidirectional craniad and caudad CSF movement signals for (A) paced breathing in the PB frequency range, (B) paced breathing in the LFO range and (c) breath holding in the LFO range. The individual waveforms show the results for a representative participant and the box plots show the group results. In group results, the purple solid line and the dotted line represents the mean and the median, the brown whiskers represent one standard deviation of the data points jittered over a 95 percent confidence interval in cream and the gray dashed line represent the threshold of statistical significance for MCCCs in the LFO range. CSF – Cerebrospinal Fluid; CSF_{Brain} – Craniad CSF movement; CSF_{Neck} – Caudad CSF Movement; CS – Chest Signal; GS – Global Signal; A.U – Arbitrary Units; MCCC – Maximum Cross-Correlation Coefficients; LFO – Low Frequency Oscillations (0.01 Hz - 0.1 Hz); PB – Paced Breathing Breathing (0.1 Hz – 0.2 Hz).

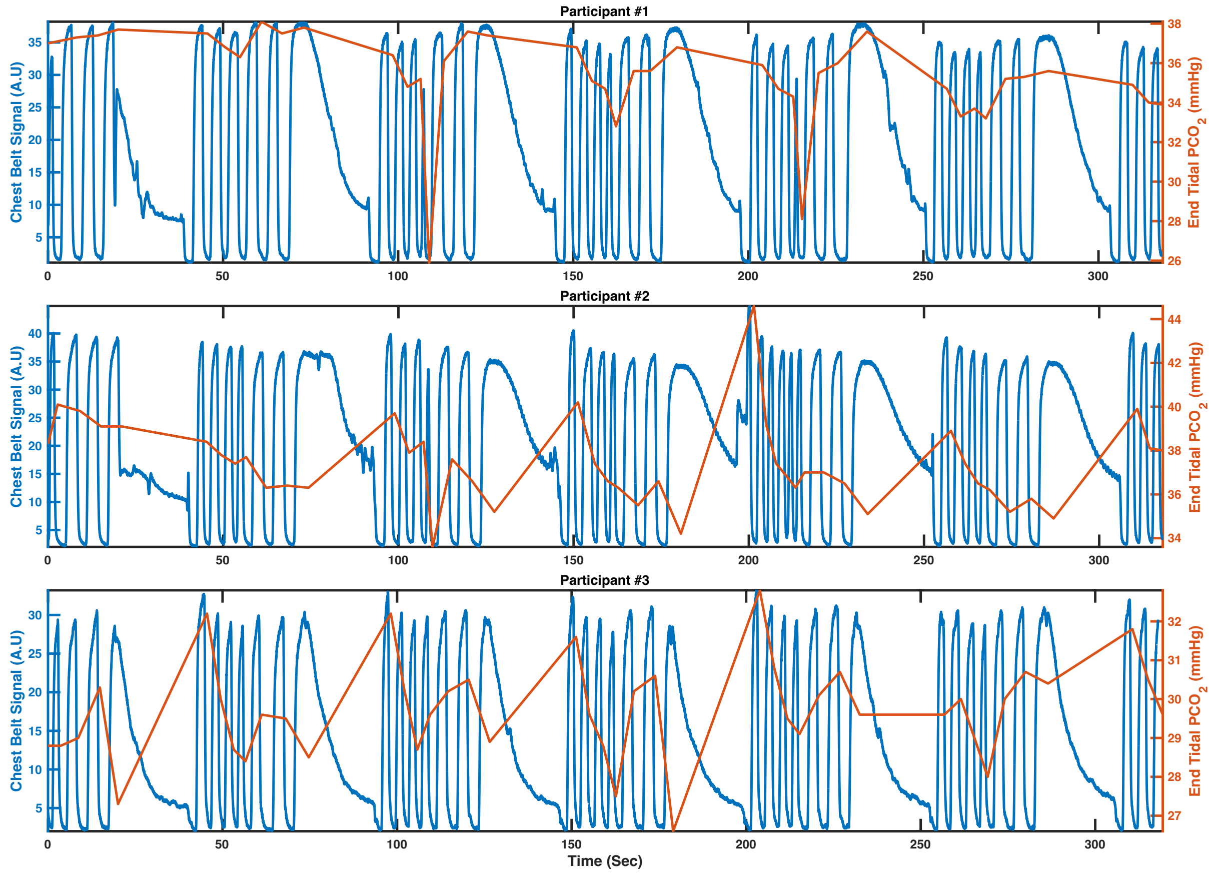

Figure S4: Temporal traces of end-tidal partial pressure of CO_2 (in red) and chest belt respiration signals (in blue) that was collected during paced breathing sessions performed at the frequency of 0.1667 Hz, as part of the data published previously from our group (Yang et al., 2020). It can be seen from this data that the end-tidal partial pressure of CO_2 decreases during paced breathing periods. On average, paced breathing at 0.1667 Hz results in an end-tidal CO_2 partial pressure decrease of 12.34±31%. Furthermore, it can also be seen that this mild hypocapnia is transient, consistent with previous literature.. A.U – Arbitrary Units; PB – Paced Breathing.

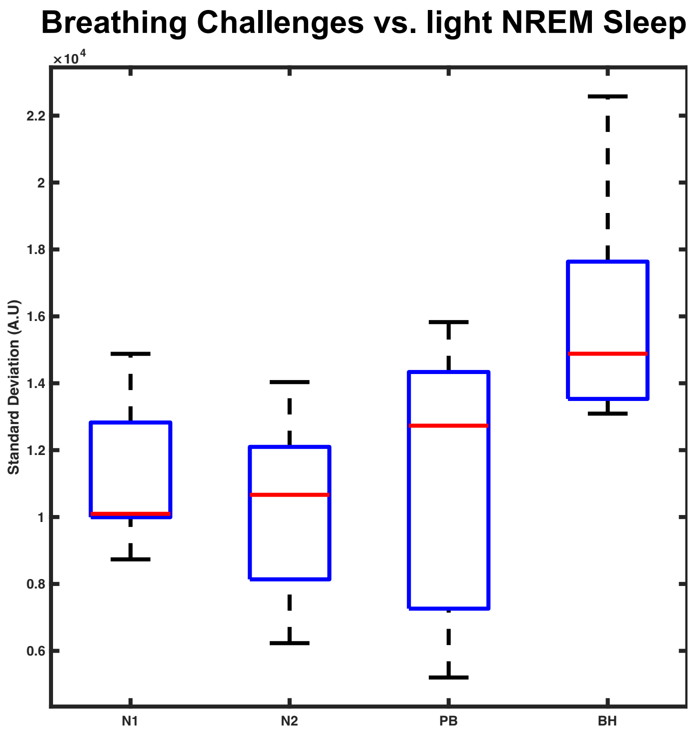

Figure S5: Box plots illustrating standard deviations of CSF movements into the brain during NREM sleep stages 1 and 2 and the biphasic CSF movements during paced breathing and breath holding. It must be noted here that this analysis used detrended CSF inflow data (not converted into velocities) for uniformity in comparison. Moreover, to account for the biphasic directionality of breathing challenge data, the standard deviations calculated from the unidirectional sleep data were multiplied by a factor of 2. Although, there are no statistical differences between the groups (as confirmed by non-parametric Kruskal-Wallis tests), the fluctuations generated by breath holding appears to be relatively larger than the light NREM sleep stages, whereas that elicited during paced breathing is comparable to that of light sleep. A.U – Arbitrary Units; CSF - Cerebrospinal Fluid; NREM – Non-Rapid Eye Movement sleep; N1 – NREM stage 1; N2 – NREM stage 2; PB – Paced breathing; BH – Breath Holding.

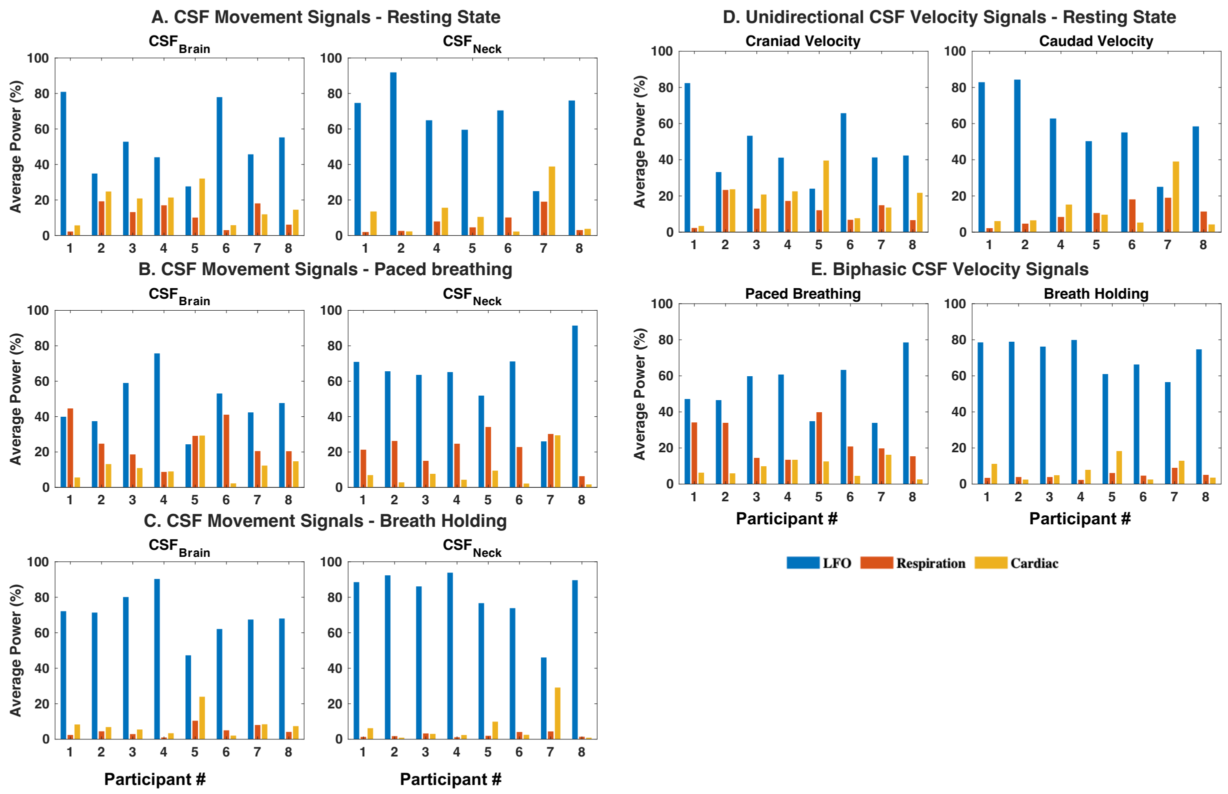

Figure S6: Power Analysis of independent CSF inflow, unidirectional CSF velocity (resting state), and biphasic CSF velocity signals during all experimental conditions. LFO: 0.01 Hz – 0.1 Hz, Respiration: 0.1 Hz – 0.2 Hz for Paced Breathing and 0.2 Hz – 0.4 Hz for all other conditions and Cardiac: 0.8 Hz – 1.13 Hz

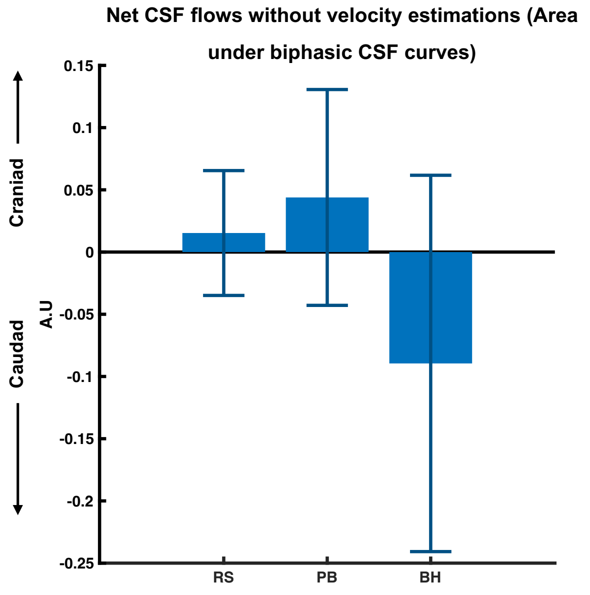

Figure S7: The net CSF volume flows estimated as area under the biphasic curves reconstructed from normalized CSF inflow signals without velocity/flowrate estimations.

Groups	Effect Size - Cohen's d	
	Net CSF Volume	CSF Amplitude Variation
RS - PB	0.25 (small)	0.29 (small)
RS - BH	1.1 (very large)	1.32 (very large)
PB - BH	0.77 (large)	1.26 (very large)

Table S2: Cohen's d values representing the effect sizes of net CSF volume flows and amplitude variations between the experimental groups. It can be seen that effect size of the net CSF volume generated by breath holding task is very large in comparison to resting state, although there is no statistical significance (p-value = 0.71). Similarly, a small effect size was revealed in the case of net CSF flow volumes generated by the paced breathing task as well, compared to resting state, even with no statistical significance (p-value = 0.73). It should also be noted that similar trends can be seen in the effect sizes of CSF amplitude variation comparisons as well. These results should be evaluated under the light that the statistical significance level cannot be used as an indicator of effect size. Statistical significance tested by p-values as well as the power of these tests depend upon sample sizes, whereas effect size is independent of them (Sullivan & Feinn, 2012). Therefore, it is possible that the data from the present study did not show statistical significance due to a relatively smaller sample size (N = 8), despite having a considerable effect size between the groups. Considering this relatively smaller sample size, effect size is a better predictor of the significance of our results. CSF – Cerebrospinal Fluid; RS- Resting State; PB – Paced Breathing; BH – Breath Holding.

References

Sullivan, G. M., & Feinn, R. (2012). Using Effect Size—or Why the P Value Is Not Enough . *Journal of Graduate Medical Education*, *4*(3), 279–282. https://doi.org/10.4300/jgme-d-12-00156.1

Yang, H. C., Liang, Z., Vike, N. L., Lee, T., Rispoli, J. V., Nauman, E. A., Talavage, T. M., & Tong, Y. (2020). Characterizing near-infrared spectroscopy signal under hypercapnia. *Journal of Biophotonics*, *13*(11), 0–3. https://doi.org/10.1002/jbio.202000173
